# Trabecular texture and paraspinal muscle characteristics for prediction of first vertebral fracture: a QCT analysis from the AGES cohort

**DOI:** 10.3389/fendo.2025.1566424

**Published:** 2025-03-26

**Authors:** Jana Hummel, Klaus Engelke, Sandra Freitag-Wolf, Eren Yilmas, Stefan Bartenschlager, Sigurdur Sigurdsson, Vilmundur Gudnason, Claus-C. Glüer, Oliver Chaudry

**Affiliations:** ^1^ Department of Medicine 3, Friedrich-Alexander-University Erlangen-Nürnberg and Universitätsklinikum Erlangen, Erlangen, Germany; ^2^ Institute of Medical Informatics and Statistics, Kiel University, Kiel, Germany; ^3^ Section Biomedical Imaging, Department of Radiology and Neuroradiology, Kiel University, Kiel, Germany; ^4^ Icelandic Heart Association Research Institute, Kopavogur, Iceland; ^5^ Institute of Radiology, Friedrich-Alexander-University Erlangen-Nürnberg and Universitätsklinikum Erlangen, Erlangen, Germany

**Keywords:** fracture prediction, vertebral fracture, computed tomography, BMD, muscle, trabecular texture

## Abstract

**Introduction:**

Vertebral fractures (VFs) significantly increase risk of subsequent fractures. Areal bone mineral density (BMD) assessed by DXA and volumetric BMD by QCT, are strong predictors of VF. Nevertheless, risk prediction should be further improved. This study used data from the Age, Gene/Environment Susceptibility Reykjavik (AGES-Reykjavik) cohort to evaluate whether trabecular texture and paraspinal muscle assessments improve the prediction of the first incident VF.

**Methods:**

CT scans of the L1 and L2 vertebrae of 843 elderly subjects; including 167 subjects with incident, VFs occurring within a 5-year period and 676 controls without fractures. Image analysis included measurement of BMD, cortical thickness and of parameters characterizing trabecular architecture and the autochthonous muscles. Fifty variables were used as predictors, including a BMD, a trabecular texture and a muscle subset. Each included age, BMI and corresponding parameters of the QCT analysis. The number of variables in each subset was reduced using stepwise logistic regression to create multivariable fracture prediction models. Model accuracy was assessed using the likelihood ratio test (LRT) and the area under the curve (AUC) criteria. Bootstrap analyses were performed to assess the stability of the model selection process.

**Results:**

96 women and 78 men with prior VF were excluded. Of 50 initial predictors, 17 were significant for women and 11 for men. Bone and texture models showed significantly better fracture prediction in women (p<0.001) and men (p<0.01) than the combination of age and BMI. The muscle model showed better fracture prediction in men only (p<0.03). Compared to the BMD model alone, LRT showed a significantly improved VF prediction of the combinations of BMD with texture (women and men) (p<0.05) or with muscle models (men only) (p=0.03) but no significant increases in AUC values (AUC women: Age&BMI: 0.57, BMD: 0.69, combined model: 0.69; AUC men: Age&BMI: 0.63, BMD: 0.71, combined models 0.73-0.77)

**Discussion:**

Trabecular texture and muscle parameters significantly improved prediction of first VF over age and BMI, but improvements were small compared to BMD, which remained the primary predictor for both sexes. Although muscle measures showed some predictive power, particularly in men, their clinical significance was marginal. Integral BMD should remain the focus for fracture risk assessment in clinical practice.

## 1 Introduction

Vertebral fractures (VF) are the most common type of osteoporotic fracture (1–3) and significantly increase the risk of subsequent vertebral and other osteoporotic fractures (4, 5). Therefore, risk prediction and prevention of VF is an important goal in osteoporosis (3, 6). Areal bone mineral density (BMD) assessed by Dual X-ray Energy Absorptiometry (DXA) and volumetric BMD assessed by Quantitative Computed Tomography (QCT) are strong predictors of VF. Standardized risk ratios of approximately 2-3 have been determined (7–9), but risk prediction still should be improved and several QCT-based strategies have been developed toward this aim. One successful approach is the determination of vertebral strength by finite element analysis (FEA) (10, 11). Another is the measurement of additional parameters from the QCT scans, such as cortical thickness, trabecular texture and paraspinal muscle characteristics (12).

This study addresses two key questions: (1) Can fracture risk prediction be improved beyond standard BMD measurement? (2) Are additional parameters that would improve fracture risk prediction are easy to measure and applicable across different CT scanners and can they be measured with precision errors of 1-2% (13). From a clinical perspective the first question is most important but from a pathophysiological perspective it may be more interesting to determine whether muscle density, muscle volume and parameters characterizing the muscle fat infiltration predict fractures independently of BMD. Of further interest are the separate contributions of trabecular and cortical bone and of the trabecular architecture to the prediction of the first incident VF.

As shown by a recent meta-analysis there is an increasing number of studies evaluating the ability of QCT to discriminate prevalent VF (14). However, prospective studies using QCT to predict incident VF are rare. A number of different analyses using FEA and lumbar and thoracic BMD parameters have been reported for the AGES-Reykjavik study (7, 15). Thoracic trabecular BMD also predicted incident VF in a large multiethnic MESA study of 6800 subjects with atherosclerosis (16). However, a multivariable approach has not been reported so far.

The relevance of paraspinal, thigh and pelvic muscles and also of soft tissue characteristics for hip (17–21), vertebral (22–24) or multiple (25) fractures has been addressed in several recent CT studies. However, most of these studies focused on the hip and most of them were cross-sectional in design with limited sample size, making the interpretation of multivariable results difficult. As summarized in a recent review (26), other studies have used magnetic resonance imaging to investigate the associations between paraspinal muscle characteristics and osteoporotic fracture, but these studies did not obtain BMD data and MR studies are too time consuming and expensive for wide spread use.

In this study we used a subset of the prospective Age, Gene/Environment Susceptibility Reykjavik (AGES-Reykjavik) study, a large epidemiologic study from Iceland (27) to compare the prognostic power of various CT assessments, including BMD, trabecular texture and paraspinal muscle characteristics in univariate and multivariable models, hypothesizing that prediction of the first incident VF occurrence based on vertebral BMD by QCT may be improved by these additional assessments. The same subset of the AGES-Reykjavik study has been analyzed previously (8), allowing to put our results in perspective with vertebral strength measurement by FEA.

## 2 Materials and methods

### 2.1 Subjects

This study utilizes a retrospective analysis of CT scans of the lumbar vertebrae L1 and L2 from a subset of subjects of the study AGES‐Reykjavik (27) of over 5,000 elderly subjects from Iceland. In summary, in a previous study (8) a case-control design was employed to select a subset of 843 subjects (497 women and 346 men). 167 subjects had sustained an incident spine fracture within a 5-year period. Spine fractures were confirmed on CT scout scans covering T6-L4, which were obtained at 5 years after baseline, using the Genant SQ scoring system (28). CT scout scans from the baseline CT scans were used to identify prior vertebral fractures, i.e., those that were already present at the time of the baseline scan. By excluding those, incident vertebral fractures were identified. The 676 subjects of the control group were randomly selected from the AGES cohort without fractures.

### 2.2 CT scanning and analysis

**Figure 1:**
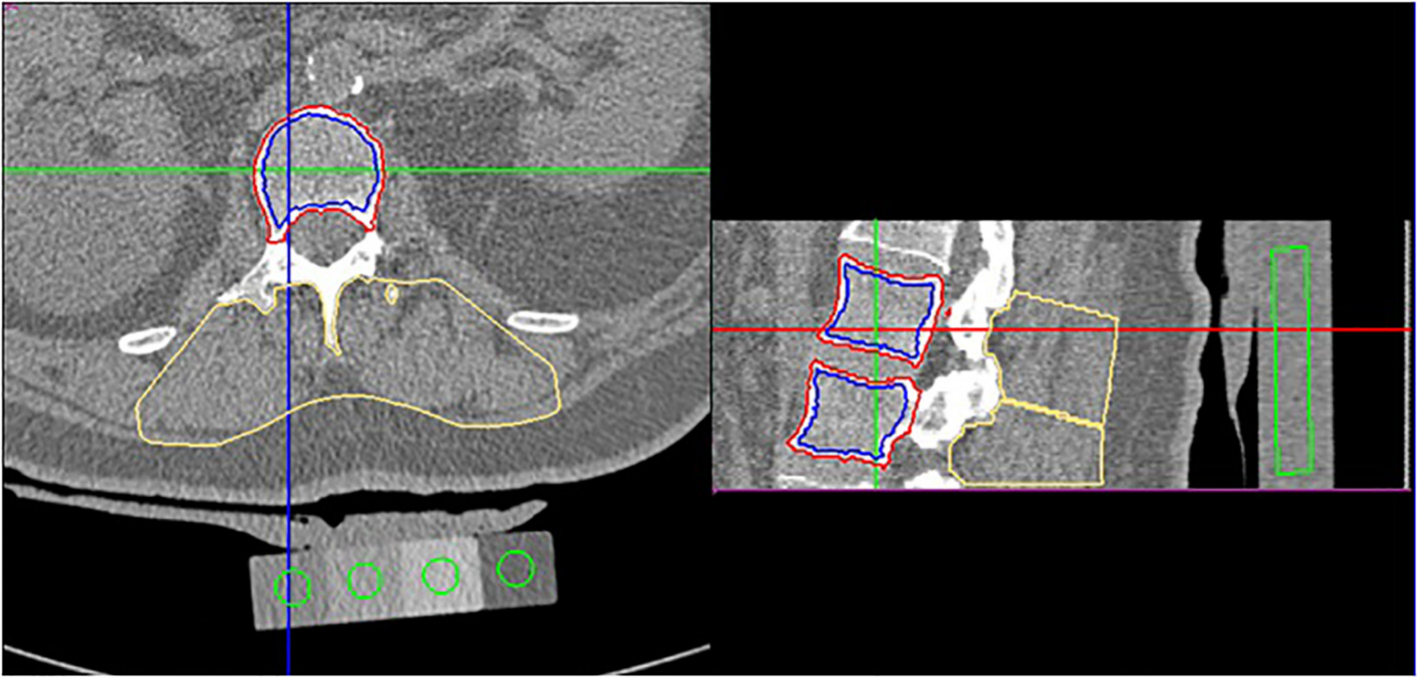
CT of the lumbar spine covering L1 and L2. The images show cropped axial and sagittal views. The green cylinders show the volumes of interest (VOIs) used to analyze the 4 different density compartments of the Image Analysis type 4 phantom. The red and blue contours delineate the integral and trabecular VOIs resulting from the 3D segmentation of L1 and L2. The yellow contours delineate the autochthonous muscle VOIs for L1 and L2.

All CT scans were performed using the same CT scanner (Sensation 4, Siemens, Erlangen, Germany) with the same CT acquisition and reconstruction protocol (120 kV, tube current modulation with 150 effective mAs, 50 cm FOV, 1 mm slice thickness, 1 mm reconstruction increment, B30s reconstruction kernel). An Image Analysis type 4 phantom (Image Analysis, Inc., Columbia, KY) was utilized for the purpose of simultaneous calibration of CT to BMD values. In the majority of subjects, the CT scan encompassed L1 and L2 vertebrae. However, in cases of fracture or other conditions that resulted in the exclusion of one of these vertebrae from the analysis, T12 and L1 or L2 and L3 vertebrae were scanned instead. The QCT analysis was performed using MIAF-Spine version 6.0.7 (**Figure 1**, **Supplementary Figure S1**). All QCT parameters that were analyzed were averaged over the two vertebrae that were covered by the CT scan. It should be noted that DXA scans were not obtained.

A comprehensive investigation was conducted, encompassing the measurement of three distinct QCT subsets (S1–S3). The first subset, designated as S1 - BMD set, involved a conventional analysis of integral, cortical, and trabecular BMD, BMC, and volume, complemented by an assessment of cortical thickness (13). The second set (S2 - texture set) involved parameters that characterized the trabecular architecture of the vertebral body. The third set (S3 - muscle set) involved parameters that characterized the autochthonous muscles at the vertebral levels present in the CT scan (**Figure 1**). A detailed description of the parameters used in this study is given in the Supplement. These muscles were not further subdivided. The psoas was not assessed because in comparison to the autochthonous muscles the percentage of intermuscular adipose tissue of the psoas is much lower and the distribution of the muscle tissue is more homogeneous. To enhance the reproducibility of the autochthonous muscle parameters, the outer edges of the muscles were excluded from the segmentation process (29). The distribution of muscle fat infiltration was subsequently measured once more via texture parameters. Further details can be found in the **Supplement (Figure S2)**.

### 2.3 Statistics

The initial data set comprised age, BMI, and 50 variables that were analyzed by MIAF-Spine. These variables served as predictors for the assessment of the first incident VF. Specifically, S1 comprised 18 predictors, S2 contained 7 predictors and S3 comprised 25 predictors. Detailed descriptions can be found in the Supplement. The z-transformation was employed to standardize all predictors. Subsequent analyses were conducted in two distinct groups: men and women. Sex-specific standard deviations of the control group were utilized for standardization purposes. Standardized age- and BMI-adjusted univariate odds ratios (OR) were calculated for each parameter.

For each subset S1-S3, stepwise logistic regression was used to obtain multivariable fracture prediction models. The initial number of predictors was reduced by minimizing the Akaike information criterion (30). The bidirectional stepwise selection was initiated with a model comprising only age and BMI, and it iteratively evaluated the inclusion or exclusion of predictors. Irrespective of their statistical significance, age and BMI were retained in all models. Other non-significant predictors (p > 0.05) were excluded. The variance inflation factor (VIF) was employed to assess multicollinearity. Predictors with VIF values greater than 5 were systematically eliminated, beginning with the predictor that exhibited the highest VIF. Subsequent to each elimination, a re-evaluation of the model ensued, resulting in the exclusion of further nonsignificant predictors. This iterative process was repeated until all VIF values were below 5, thereby ensuring minimal collinearity among the final predictors of each subset’s model.

The BMD model S1 was selected as the reference model. Significant predictors from another subset model, e.g. the muscle model, were added to S1 to create combined models. The fracture prediction of the combined models was compared with that of S1 using nested logistic regression following the approach suggested by Harrell (30). To ascertain whether the combined model significantly improved fracture prediction compared to S1, the likelihood ratio test (LRT) was used. The LRT adheres to a chi-squared (χ^2^) distribution and provides p-values for the comparison of nested models. Receiver operator characteristic (ROC) curves and their area under the curve (AUC) values, also used as performance metric, were compared using bootstrap confidence intervals (CI) and tests (31).

The same procedure was applied to compare fracture prediction of individual subsets S1-S3 with that of age and BMI. To assess the stability of the stepwise model selection process, a bootstrap analysis was performed with 1,000 resampled data sets. For each bootstrap sample, the stepwise procedure was repeated, and the frequency of predictor inclusion in the resulting models was recorded. Furthermore, the AUC values were calculated for each bootstrap iteration to assess the variability of model performance.

Finally, the combined models were also calculated in women with fracture SQ grades of 2 and 3, thereby excluding the mild SQ 1 fractures. All statistics were performed using R (R Core Team, version 4.3.2, functions ‘stepAIC’ [package: MASS] and ‘roc.test’ [package: pROC]).

## 3 Results

**Figure 2:**
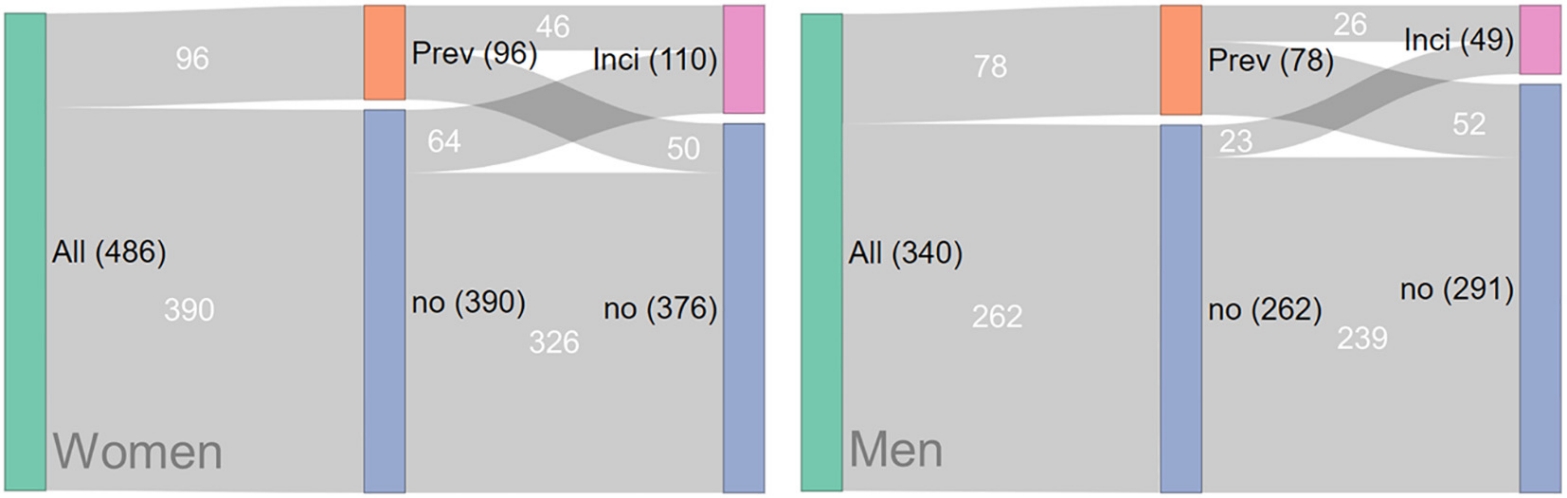
Sankey plots illustrating the populations of female and male subjects with prevalent, incident, and no vertebral fractures (VF). QCT images corresponding to the central blue and orange bars were available, while fracture status information for the right blue and pink bars was also included. Black numbers indicate the number of patients in each bar, while white numbers denote the contributions from other bars. It is important to note that subjects with prevalent fractures (orange) were excluded from the analysis in this study; thus, only patients represented in the central blue bar (controls and those with first incident VF) were included for analysis.

A total of 826 CT data sets (486 women and 340 men) of the original subsample of 843 subjects were analyzed. The analysis of CT scans from 17 subjects was not possible, for the majority of cases due to the presence of excessive osteophyte formation and substantial bone sclerosis in the vicinity of the endplates. At baseline, 96 of the 486 women and 78 of the 340 men had prior VF (**Figure 2**). These subjects were excluded from the analysis of the current study with the objective of determining the risk prediction of the first incident VF.

Patient characteristics and significant univariate predictors for fracture occurrence in women are shown in **Table 1** and in men in **Table 2**. All univariate ORs were adjusted for age and BMI, which are also included in the aforementioned tables. In women, 17 of the initial 50 predictors were found to be significant predictors of future fractures, while in men, 11 of the initial 50 predictors were found to be significant. The non-significant predictors (p < 0.05) are not displayed in the tables.

**Table 1:** Significant univariate predictors of the first incident vertebral fracture in women.

		Controls	Incident VF					
	n	326	64					
		Mean ± SD		AUC	CI	OR/SD	CI	p
	Age [y]	73.9 ± 5.1	75.2 ± 5.6	0.57	(0.49; 0.64)	0.79	(0.6; 1.0)	0.07
	BMI [kg/m^2^]	27.6 ± 4.5	27.5 ± 5.2	0.51	(0.43; 0.60)	1.03	(0.8; 1.3)	0.83
Subset	Predictor	Mean ± SD		AUC	CI	OR/SD	CI	p
S2-Texture	Trab_gInhomo	73.9 ± 5.1	75.2 ± 5.6	0.66	(0.59; 0.73)	2.07	(1.4; 3.1)	< 0.001
S1-BMD	BMD_Int_tVB [mg/cm^3^]	175.7 ± 37.5	156 ± 33.9	0.67	(0.60; 0.74)	1.86	(1.3; 2.6)	< 0.001
S1-BMD	BMD_Trab_mCy [mg/cm^3^]	83.7 ± 33.3	67.6 ± 27.6	0.66	(0.59; 0.73)	1.84	(1.2; 2.7)	< 0.01
S1-BMD	BMC_Int_tVB [g]	5.6 ± 1.25	4.95 ± 1.08	0.67	(0.59; 0.74)	1.84	(1.3; 2.6)	< 0.001
S2-Texture	Trab_Vario_slope	6.1 ± 1	5.8 ± 0.8	0.62	(0.55; 0.70)	1.83	(1.2; 2.8)	< 0.01
S1-BMD	BMD_Trab_tVB [mg/cm^3^]	90.8 ± 30.5	75.8 ± 28.6	0.66	(0.59; 0.73)	1.79	(1.2; 2.5)	< 0.01
S1-BMD	BMD_Trab_cCy [mg/cm^3^]	77.9 ± 29.5	64.2 ± 25.5	0.65	(0.58; 0.72)	1.78	(1.2; 2.6)	< 0.01
S1-BMD	BMC_Trab_tVB [g]	1.72 ± 0.56	1.45 ± 0.5	0.65	(0.58; 0.72)	1.74	(1.2; 2.4)	< 0.01
S1-BMD	Thick_Cort_LE [mm]	1.06 ± 0.13	1.02 ± 0.07	0.62	(0.55; 0.69)	1.70	(1.1; 2.5)	< 0.01
S1-BMD	BMC_Cort_tVB [g]	2.79 ± 0.7	2.48 ± 0.61	0.65	(0.58; 0.72)	1.70	(1.2; 2.4)	< 0.01
S1-BMD	BMD_Cort_tVB [mg/cm^3^]	367.1 ± 52.9	342.4 ± 51.2	0.65	(0.57; 0.73)	1.64	(1.2; 2.2)	< 0.01
S1-BMD	BMC_Cort_mVB [g]	0.62 ± 0.2	0.54 ± 0.17	0.64	(0.56; 0.71)	1.62	(1.1; 2.3)	< 0.01
S1-BMD	Thick_Cort_tVB [mm]	1.31 ± 0.18	1.25 ± 0.14	0.62	(0.55; 0.70)	1.58	(1.1; 2.2)	< 0.01
S1-BMD	BMD_Cort_mVB [mg/cm^3^]	403.1 ± 63	377 ± 60.6	0.64	(0.56; 0.72)	1.53	(1.1; 2.1)	0.010
S1-BMD	Thick_Cort_mVB [mm]	1 ± 0.28	0.89 ± 0.24	0.63	(0.55; 0.71)	1.50	(1.1; 2.1)	0.013
S1-BMD	Vol_Cort_mVB [cm^3^]	1.5 ± 0.29	1.39 ± 0.26	0.62	(0.55; 0.70)	1.48	(1.1; 2.1)	0.012
S1-BMD	Vol_Cort_tVB [cm^3^]	7.53 ± 1.2	7.16 ± 1.02	0.61	(0.53; 0.68)	1.43	(1.1; 1.9)	0.025

Mean ± SD of univariate predictors with area under curve (AUC) values and their confidence intervals (CI), all values are sorted by Odds ratios (OR). OR are calculated per one standard deviation decrease. AUC and OR values are adjusted for age and BMI, n.s. predictors are not listed here. CI gives the confidence interval of OR and p the significance level. A detailed description of the parameters is given in the Supplement.

**Table 2:** Significant univariate predictors of the first incident vertebral fracture in men.

		Controls	Incident VF					
	n	239	23					
		Mean ± SD		AUC	CI	OR/SD	CI	p
	Age [y]	74.4 ± 5.1	76.3 ± 4.8	0.62	(0.51; 0.73)	0.71	(0.5; 1.1)	0.10
	BMI [kg/m^2^]	26.8 ± 3.6	26.0 ± 3.4	0.58	(0.45; 0.71)	1.27	(0.8; 2.0)	0.31
Subset	Predictor	Mean ± SD		AUC	CI	OR/SD	CI	p
S1-BMD	BMD_Int_tVB [mg/cm^3^]	195.5 ± 38.9	169.1 ± 34.7	0.71	(0.61; 0.82)	2.05	(1.2; 3.5)	< 0.01
S1-BMD	BMD_Trab_tVB [mg/cm^3^]	104.8 ± 29.7	84.6 ± 27.2	0.70	(0.59; 0.82)	2.02	(1.2; 3.4)	< 0.01
S1-BMD	BMC_Trab_tVB [g]	2.79 ± 0.83	2.24 ± 0.74	0.71	(0.59; 0.82)	2.01	(1.1; 3.5)	0.012
S1-BMD	BMD_Cort_tVB [mg/cm^3^]	411.7 ± 59.4	372.7 ± 48.6	0.71	(0.61; 0.82)	1.98	(1.2; 3.3)	< 0.01
S1-BMD	BMC_Int_tVB [g]	8.45 ± 2	7.27 ± 1.56	0.69	(0.58; 0.80)	1.96	(1.1; 3.5)	0.017
S1-BMD	BMD_Cort_mVB [mg/cm^3^]	457.5 ± 69.9	413.9 ± 57.4	0.70	(0.60; 0.80)	1.92	(1.1; 3.2)	0.011
S1-BMD	BMD_Trab_cCy [mg/cm^3^]	87.5 ± 28.8	69.2 ± 26.6	0.70	(0.58; 0.81)	1.83	(1.1; 3.1)	0.020
S1-BMD	BMD_Trab_mCy [mg/cm^3^]	93 ± 34.1	73.8 ± 30.6	0.69	(0.58; 0.80)	1.72	(1.0; 2.9)	0.042
S3-Muscle	M_gAniso_Bin6	57.3 ± 0.2	57.4 ± 0.2	0.69	(0.60; 0.78)	0.61	(0.4; 1.0)	0.031
S2-Texture	Diff_Box_C	2.65 ± 0.04	2.66 ± 0.04	0.68	(0.58; 0.79)	0.48	(0.3; 0.9)	0.022
S2-Texture	Trab_lAniso	69.3 ± 1.9	69.7 ± 1.8	0.69	(0.59; 0.80)	0.46	(0.2; 0.8)	0.013

Mean ± SD of univariate predictors with area under curve (AUC) values and their confidence intervals (CI), all values are sorted by Odds ratios (OR). OR are calculated per one standard deviation decrease. AUC and OR values are adjusted for age and BMI, n.s. predictors are not listed here. CI gives the confidence interval of OR and p the significance level. A detailed description of the parameters is given in the Supplement.

In both sexes, a trabecular texture predictor demonstrated the numerically highest OR for the first incident VF. However, the confidence intervals of ORs for all significant predictors largely overlapped. It is noteworthy that among women, no muscle parameters exhibited statistically significant ORs for the first incident VF, while among men, only one muscle parameter demonstrated a statistically significant OR for the first incident VF. However, the means of this predictor did not differ significantly between male control and fracture cases (p = 0.42).

**Table 3**; **Supplementary Tables S2, S3** present the results of the subset-specific stepwise logistic regressions. The AUC results are presented in **Table 3**. The predictors that remained in the S1, S2 and S3 models are listed in **Supplementary Tables S2, S3**. In addition to age and BMI, in the final models only one or two predictors remained of each subset, indicating a high correlation among the parameters analyzed of a given subset. For the sake of comparison, **Table 3** also shows results of the model of age and BMI. From LRT results, the models based on S1 and S2 exhibited significantly (p ≤ 0.01) higher fracture prediction than the combination of age and BMI alone (**Table 4**). This was also the case for S3 in men (p = 0.03) but not in women. Combinations of S2 and S3 models with S1 showed a significant improvement in VF prediction compared to S1 alone (p < 0.05), except for S3 in women.

**Table 3:** AUC values for a combination of age and BMI and for the subset specific models (that are also adjusted for age and BMI).

	Women		Men	
	AUC	CI	AUC	CI
Age & BMI	0.57	(0.49; 0.65)	0.63	(0.51; 0.74)
S1 BMD	0.69	(0.62; 0.76)	0.71	(0.60; 0.82)
S2 Texture	0.67	(0.59; 0.75)	0.72	(0.62; 0.83)
S3 Muscle	*		0.69	(0.60; 0.79)

*No predictors of S3-Muscle remained in the final model.

**Table 4:** Performance of combinations of nested models tested by LRT: Model 1, which is the base model, and Model 2, which represents the combined model.

Comparison of Nested Models		Women			Men		
Model 1	Model 2	DoF	χ2	p	DoF	χ2	p
Age & BMI	S1 BMD	385	18.5	<0.001	259	6.4	0.01
Age & BMI	S2 Texture	386	15.0	<0.001	258	6.9	0.01
Age & BMI	S3 Muscle	*			258	4.8	0.03
S1 BMD	S2 Texture	385	5.9	0.02	258	4.5	0.05
S1 BMD	S3 Muscle	*			258	4.8	0.03

*No predictors of S3-Muscle remained in the final model.

**Figure 3:**
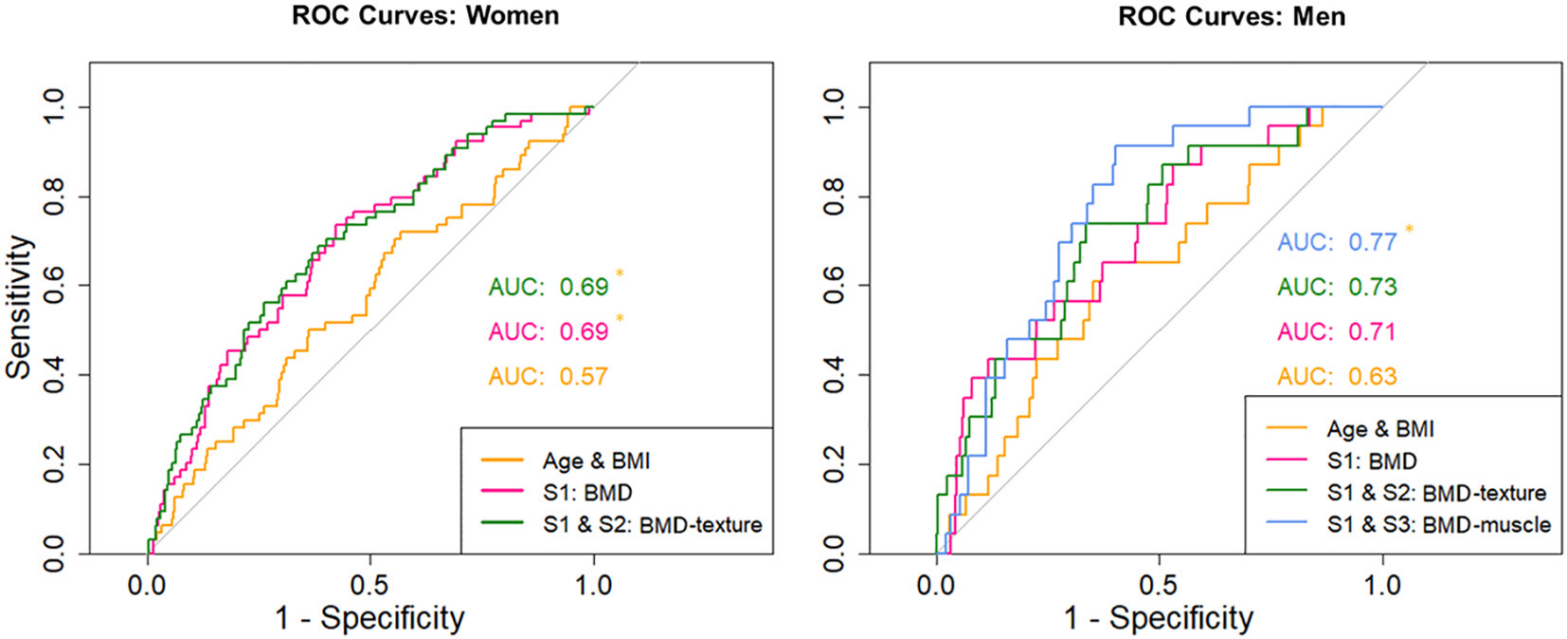
Receiver operator curves for women - for age/BMI, S1 and combinations of S1 with S2. Receiver operator curves for men - for age/BMI, S1 and combinations of S1 with S2 and S1 with S3. Asterisk marks significant difference (p < 0.05) in the AUC values against the model only including age and BMI.

ROC curve plots are summarized in **Figure 3**. For women, AUC values of S1 (0.69) and of the S1-S2 combination (0.69) were significantly (p < 0.05) higher than for the combination of age and BMI (0.57) but AUC values of the S1-S2 combination were not significantly higher than for the S1 model. For men, only the S1-S3 combination (0.77) was significantly (p < 0.05) higher than the combination of age and BMI (0.63), no other significant differences were detected.

Integral BMD of the vertebral body remained a significant predictor for both men and women in S1, the reference model utilized in this study. In women, the cortical thickness of the lower endplate also persisted as a significant predictor in S1. The bootstrap procedure demonstrated that, including age and BMI, on average 5.4 (CI 3-9) predictors remained significant in women, with a mean AUC of 0.72 (CI 0.65 – 0.79). In men, an average of 5.7 (CI 3-11) predictors remained significant, with a mean AUC of 0.8 (CI 0.71 – 0.92). The frequency of predictors that remained significant in each of the 1,000 resampled datasets is documented in **Supplementary Table S4**.

For the sake of comparison, the AUC values were also calculated for a manually selected model based on clinical expertise. This model, in addition to age and BMI, consisted of integral BMD of the total vertebral body (BMD_Int_tVB) and cortical thickness measured at the midsection of the vertebral body (Thick_Cort_mVB). However, the latter variable did not emerge as a significant predictor in the previous analysis for both men and women. The AUC results were found to be 0.67 (CI 0.60 – 0.74) for women and 0.72 (CI 0.61 – 0.83) for men.

In women, 34 first incident VFs were diagnosed with SQ 1, while an additional 30 were diagnosed with SQ 2 or SQ 3. Excluding SQ 1 fractures, the AUC values increased to 0.72 for S1, 0.7 for S2, and 0.73 for S3 compared to the values in **Table 3**. The LRT of S3 exhibited a borderline significant increase (p = 0.05) in comparison to that of age and BMI. The performance of the combined models is shown in **Supplementary Table S5**. In contrast to the subset encompassing SQ1 to SQ3 fractures, the combination of S2 and S1 no longer was statistically superior in comparison to S1. However, the incorporation of a muscle predictor enhanced the prediction of VF, surpassing the performance of S1. The AUC values remained significantly different for the comparison against age and BMI. For men there were too few cases to perform such an analysis.

## 4 Discussion

In this study, the performance of 50 parameters obtained from QCT scans of the spine was assessed to predict the first incident VF univariately or in combination. Volumetric BMD based models significantly predicted the first incident VF with AUCs at about the same level as those reported for FEA in previously published studies on a sample very similar to ours (8). The analysis revealed that independent of BMD, parameters of trabecular texture and with limitations also of autochthonous muscle significantly improved the prediction of vertebral fractures (VF), compared to age and BMI alone. However, when compared to BMD, the enhancement was minimal and likely to be of negligible clinical significance. Thus age and BMI adjusted volumetric BMD, that can easily be measured with QCT and with excellent precision is the parameter of choice for prediction of incident fractures in clinical routine.

QCT of the spine is typically used to measure average trabecular and integral BMD of L1 and L2 (32). Therefore, the reference model (S1) extracted from 18 different BMD and cortical thickness measures was used as ‘QCT gold standard’ for prediction of the first incident VF. After the reduction of variables in the stepwise logistic regression, only integral BMD of the vertebral body remained for both sexes and in addition, cortical thickness of the lower endplate for women. As age and BMI alone are important predictors of incident VF and in order to be consistent with most publications on fracture prediction that typically report age and BMI adjusted risk ratios or AUC values, age and BMI were retained in all models, even if these two parameters were not significant in the regression step.

The term ‘gold standard’ implies that there is an optimum set of variables that should be used for fracture prediction. However, the bootstrap analysis demonstrated that S1 models with different predictor combinations exhibited average AUCs that were analogous to the reference S1 model utilized in this study. It is noteworthy that parameters of cortical thickness were more frequently incorporated into the models resulting from the bootstrap process than BMD. However, with the exception of cortical thickness of the lower endplate, none of the 18 input predictors occurred in more than 30% of the 1,000 models. While it is unlikely that the S1 reference model of this study overestimated fracture risks due to overfitting, a common problem in multivariable analyses, there is no unique best set of S1 QCT variables to be used for fracture prediction. Conducting a separate analysis of a distinct subset of the AGES population, or even a different study, is likely to yield a different S1 reference model. This phenomenon is also evident in the univariate results, where the adjusted ORs for many variables were found to be highly comparable, despite adjustments for age and BMI.

From a clinical perspective, this is favorable news because a combination of rather esoteric predictor combinations will most likely not predict the first incident VF risk better than a standard set of predictors. Integral BMD, a variable that can easily be measured with high precision (13), is an adequate predictor of incident VF. Cortical thickness of the lower endplate may more reflect sclerotization of the trabecular bone due to vertebral disk impairments than actual cortical thickness of the endplate. Segmentation in this case is challenging and disk impairments were frequent in the AGES population. Nevertheless, the observation that degenerative features of the vertebrae may also be predictive of the first incident VF should be further pursued.

Therefore for the handpicked S1 model cortical thickness of the mid vertebral body was selected, which is less affected by degenerative changes (33–35). AUC values of the handpicked model were well in the range of the bootstrapping results. The addition of mid cortical thickness did only marginally improve VF prediction compared to integral BMD alone.

A notable finding is the observation that the S2 texture model predicted VF independently of the S1 BMD model. In scenarios where a BMD assessment is not feasible, for example in MRI scans, VF prediction is still possible using parameters of trabecular texture, at least in principle. Recent studies have shown that an MRI based texture analysis can be used to discriminate subjects with and without prevalent vertebral fractures (36, 37). However, it is important to remember that texture assessments depend on noise and spatial resolution (38). Thus, MRI texture results will vary significantly among MRI sequences. It should also be noted that all scans analyzed in this study were obtained from the same CT scanner using the same CT protocol. Texture measurements from different scanners may not be directly comparable.

Several other studies have demonstrated the ability of histomorphometry or texture parameters to improve the discrimination of vertebral fractures when compared with BMD (39–43), but none have investigated the ability to predict incident osteoporotic vertebral fractures. Therefore, it is an important finding of this study that texture parameters can be used to predict incident vertebral fractures. Of course, there are many different texture parameters and a radiomics approach may more systematically exploit the potential of texture parameters than the heuristic approach chosen in this study.

A substantial body of research has demonstrated a correlation between muscle metrics and spinal fractures (26, 44, 45). However, the majority of these studies were cross-sectional in design, investigating the associations of muscle metrics with prevalent conditions rather than the prediction of incident VF. In this study the predictive value of paraspinal muscle characteristics was weak, questioning their utility in clinical practice. Muscle parameters significantly predicted VF in women only after excluding the SQ 1 mild fractures and even then, the improvement of fracture prediction compared to age and BMI was only borderline significant. This finding is particularly noteworthy given the comprehensive array of parameters that were examined, encompassing muscle density, fat fraction, and a multitude of texture parameters that characterized the distribution of muscle tissue and intermuscular adipose tissue. In men, a modest effect was observed for muscle tissue anisotropy. However, the clinical interpretation of this finding is challenging, as the anisotropy did not differ significantly between men with and those without incident VF.

In the event of confirmation, the implications are substantial. The role of paraspinal muscle exercise in preventing vertebral fractures remains uncertain. Actually, a recent 12-month study in men demonstrated that exercise had no effect on paraspinal muscles, despite significant training effects on spinal BMD and thigh muscle parameters (46). Further research is needed to determine whether muscle deterioration is a cause or a consequence of fractures.

The multivariable analysis is a big advantage of this study. Instead of just presenting univariate odds or hazard ratios after adjustment for age and BMI (7, 8) the advanced statistical approach of comparing nested combinations of predictors provided the possibility to compare the performance of fracture prediction of different models. The use of the log-likelihood ratio as performance criterion guarantees statistical rigor in identifying the set of predictors that best fit the pattern of incident fractures (30, 47) but beyond the result whether fracture prediction differs, the clinical interpretation of the magnitude of improvement of fracture prediction is difficult. Therefore, we also calculated AUC values as established performance characteristic, which, however, offers less statistical power to test which model is better than others.

As shown in **Table 4** and **Supplementary Table S5** compared to S1 the inclusion of S2 predictors, which characterize trabecular architecture significantly improved prediction of VF in men and women. However, in women this was no longer the case once mild fractures were excluded. Compared to S1 the inclusion of S3 predictors, which characterize muscle significantly improved prediction of VF in men and after exclusion of SQ 1 fractures also in women. However, the ROC graphs show that AUC values of the combined models did not significantly increase AUC values. Thus, the clinical benefit is rather limited and may not be worth the effort of an advanced QCT analysis. It is a limitation of the study that the number of incident vertebral fractures with grade 2 or 3 was too small in men to perform a separate analysis.

It is another limitation of this study that FEA was not performed and therefore it was not possible to test whether a strength determination would have increased fracture prediction beyond that of BMD. Such an analysis was also not performed in the earlier study that analyzed the same cohort (8). While in that study strength showed the highest OR for fracture prediction, integral BMD was not measured and CI of the OR largely overlapped. OR calculation may be strongly affected by the distribution of the data but no test of normal distribution has been reported in the earlier study. Judging the performance based on ROC analysis showed our QCT results at the same level as the FEA data reported earlier Whether from clinical perspective the advanced method of FEA is worth the additional effort compared to a standard QCT analysis still has to be determined.

While our results do not provide a definitive solution for predicting the first incident VF, they offer valuable insights that may guide future advancements in addressing this inherently complex and unresolved challenge. Surprisingly even with our comprehensive analysis of texture, muscle and bone parameters, none of the parameters or a combination of parameters gave an outstanding improvement over established predictors, namely age and BMI adjusted volumetric BMD. Even muscle parameters that are known to perform well in cross-sectional studies did not perform extraordinary for the prediction of incident VF. The analyzed dataset is exceptionally rare and one of the few that enable such an in-depth analysis. Unfortunately, less than a handful of datasets exist for the prospective analysis of VF using QCT. Nevertheless, it would be highly valuable to validate these findings in future studies.

## Data Availability

The datasets presented in this article are not readily available because they were used under license. Restrictions also apply to the availability of data generated or analyzed during this study to preserve patient confidentiality. Data from the AGES-Reykjavik study are available through collaboration under a data usage agreement with the Icelandic Heart Association. Requests to access the datasets should be directed to AGES_data_request@hjarta.is.
