# Supplementary material for: Trabecular texture and paraspinal muscle characteristics for prediction of first vertebral fracture: a QCT analysis from the AGES cohort
Source: Front Endocrinol (Lausanne). 2025 Mar 26;16:1566424. doi: 10.3389/fendo.2025.1566424 (PMC11978659; doi:10.3389/fendo.2025.1566424)
Supplement: Supplementary file 1 [file DataSheet1.docx]

Trabecular Texture and Paraspinal Muscle Characteristics for Prediction of First Vertebral Fracture: A QCT Analysis from the AGES Cohort - Supplement

# Variables used for incident vertebral fracture predictions

Apart from age and BMI all variables were divided into three subsets containing predictors related to BMD, trabecular bone texture, and autochthonous muscle

- 1. **BMD subset**

The BMD subset included BMD, BMC and Volume assessments of various bone compartments and cortical thickness as shown in Table S 1, VOIs are visualized in Figure S1. In the paper predictor names followed the following conventions: The measured value was appended by the abbreviations of the bone compartment and the volume of interest, e.g. BMD_Int_tVB, or Vol_Cort_mVB, or Thick_Cort_uE

- 1. **Trabecular texture subset**

The following texture parameters were determined from the distribution of the BMD values in the trabecular compartment of the total vertebral body

1. Global inhomogeneity (Trab_gInhomo)
2. Local inhomogeneity (Trab_lInhomo)
3. Global anisotropy (Trab_gAniso)
4. Local anisotropy (Trab_lAniso)
5. Variogram slope (Trab_Vario_slop)
6. Entropy (Trab_Entrop)
7. Differential Box Counting (Trab_Diff_Box)

This specific selection was mostly made from the perspective of medical interpretability. Inhomogeneity is the same as BMD standard deviation, anisotropy characterizes the directedness of the trabeculae an important feature as most trabecular in the lumbar vertebrae are aligned in horizontal or vertical direction, The variogram slope is the basis of the so-called trabecular bone score (TBS) used in DXA to predict spinal fractures [1]. We investigated the dependence of the first seven parameters on noise and spatial resolution previously [2, 3]. Differential box counting, was added as a fractal measure.

- 1. **Muscle subset**

The following parameters were determined from the segmented combined left and right autochthonous muscles.

1. Muscle volume (Mus_Vol)
2. Muscle density (Mus_HU) determined as average CT value
3. Percent muscle tissue (%MT) determined as percent of volume with a CT value > 48
4. HU

Then a histogram analysis of the CT values of the segmented autochthonous muscles was performed to divide the muscle into 6 partitions (Bins): Bin 1: all voxels of pure adipose tissue and Bin 6: all voxels of pure muscle tissue (MT)(defined as HU > 48). Bins 2, 3, 4 and 5 consisted of voxels representing up to 25%, 50%, 75% and 100% MT, accordingly (Figure S2). In order to assess the adipose tissue distribution of the autochthonous muscles, the following predictors were used:

1. Percent muscle tissue (%MT) in each bin denoted as %MT_Bin1 to %MT_Bin6
2. Mean HU value of Bin 1 and Bin 6 (Mus_HU_Bin1, Mus_HU_Bin6)
3. The following texture parameters determined for Bin 1 and Bin 6
   1. Global inhomogeneity (M_gInhomo_Bin1, M_gInhomo_Bin6)
   2. Local inhomogeneity (M_lInhomo_Bin1, M_lInhomo_Bin6)
   3. Global anisotropy (M_gAniso_Bin1, M_gAniso_Bin6)
   4. Local anisotropy (M_lAniso_Bin1, M_lAniso_Bin6)
   5. Variogram slope (M_Vario_slop_Bin1, M_Vario_slop_Bin6)
   6. Entropy (M_Entrop_Bin1; M_Entrop_Bin6)
   7. M_Differential Box Counting (Diff_Box_C_Bin1, Diff_Box_C_Bin6)

# Figures

Figure S1: An overview of the different volumes of interest (VOI) used to measure BMD. The measures are shown differently for L1 and L2, but they are applicable to both vertebrae. The total vertebral body VOI (tVB) is shown in red (with the trabecular VOI in dark blue). The central cylinder (cCy) VOI and its mid-section (mCy) are shown in cyan in L1, in both sagittal and coronal views. In L2, the upper vertebral endplate (uE) VOI is shown in yellow, while the lower endplate (lE) VOI is shown in green. The mid-vertebral body VOI is defined as the volume between the upper and lower endplates (between the yellow and green borders), which is also shown in the sagittal and coronal views.


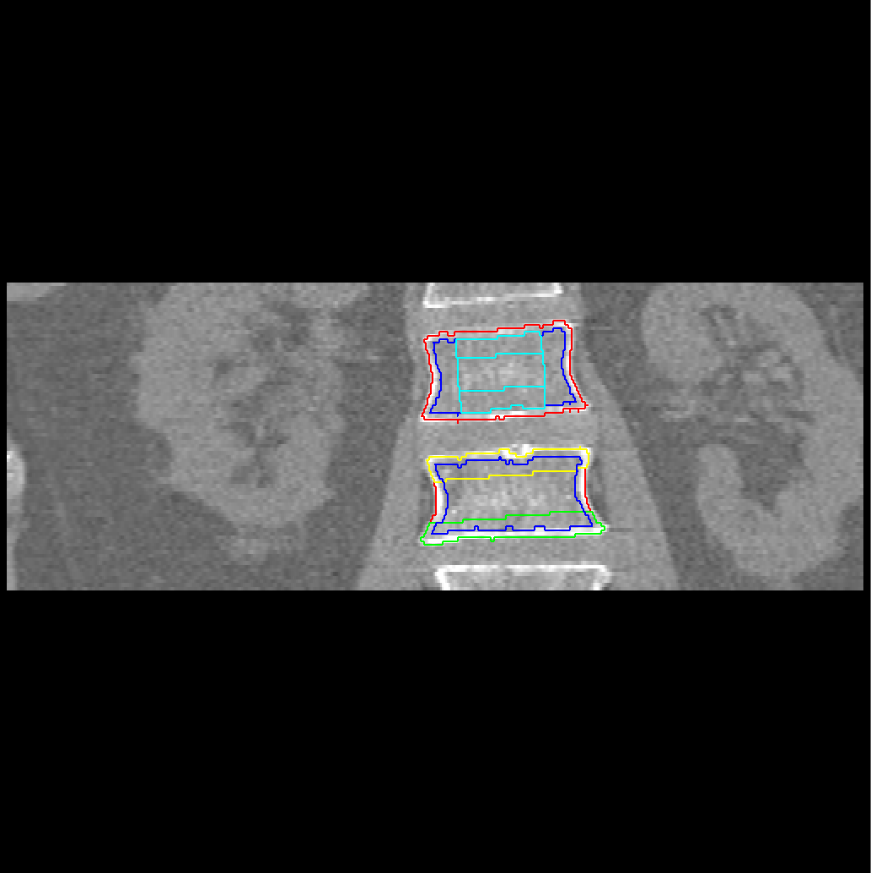

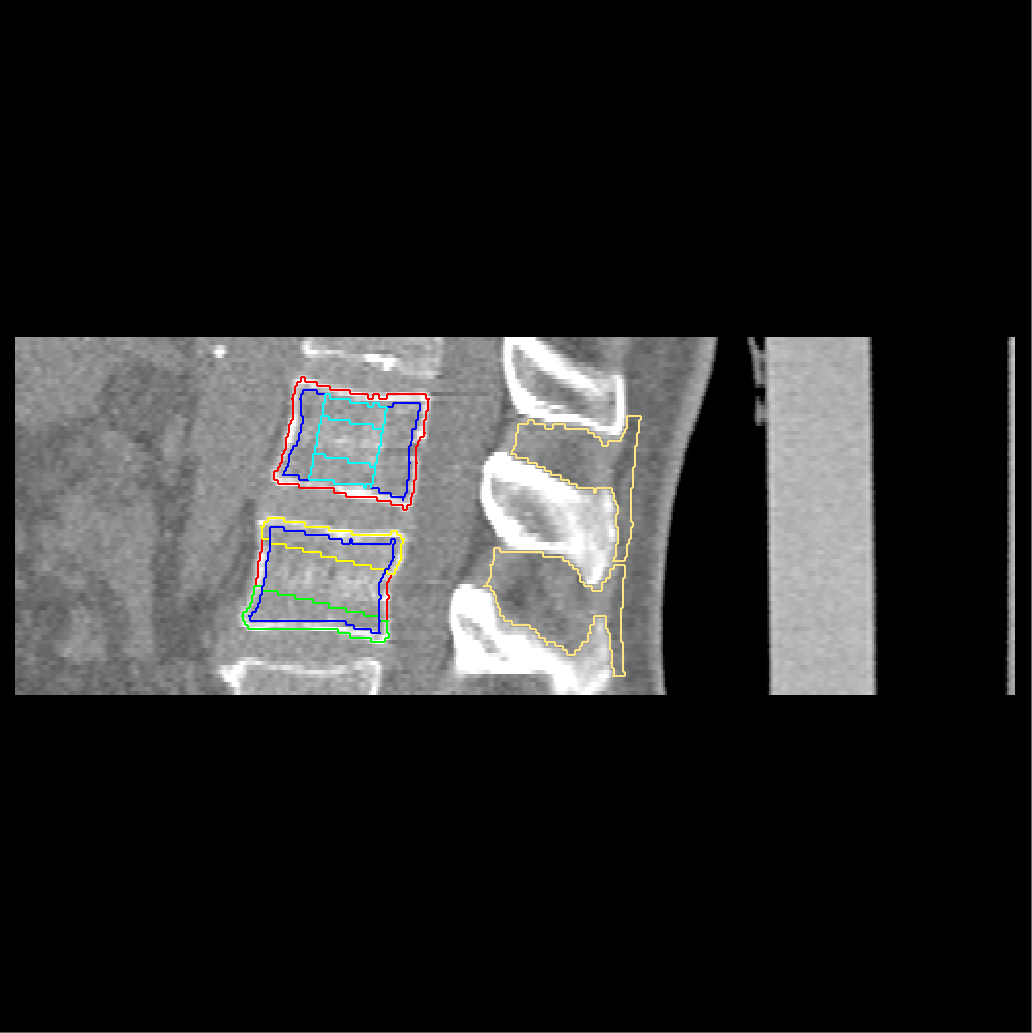

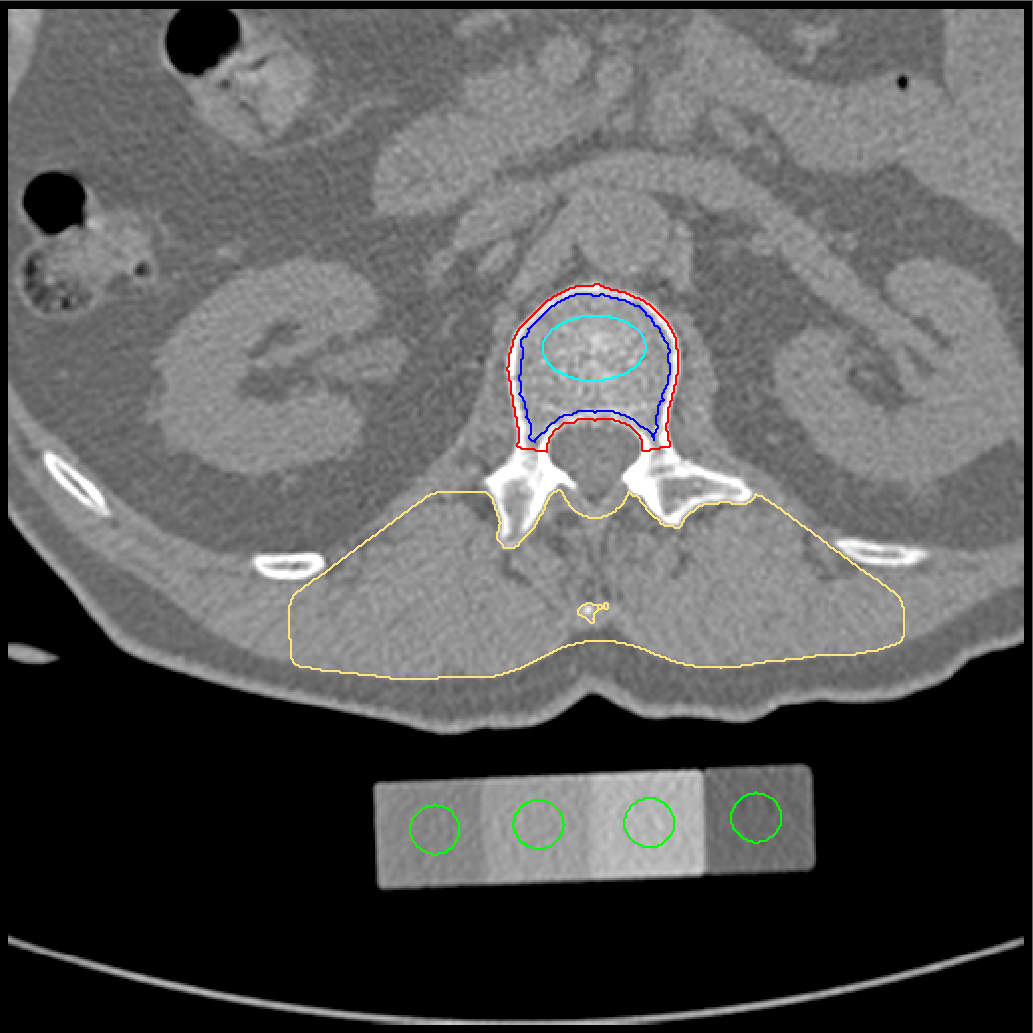


Figure S2: Left: segmentation of muscle VOI; right: histogram of CT values of muscle VOI and partition into 6 bins


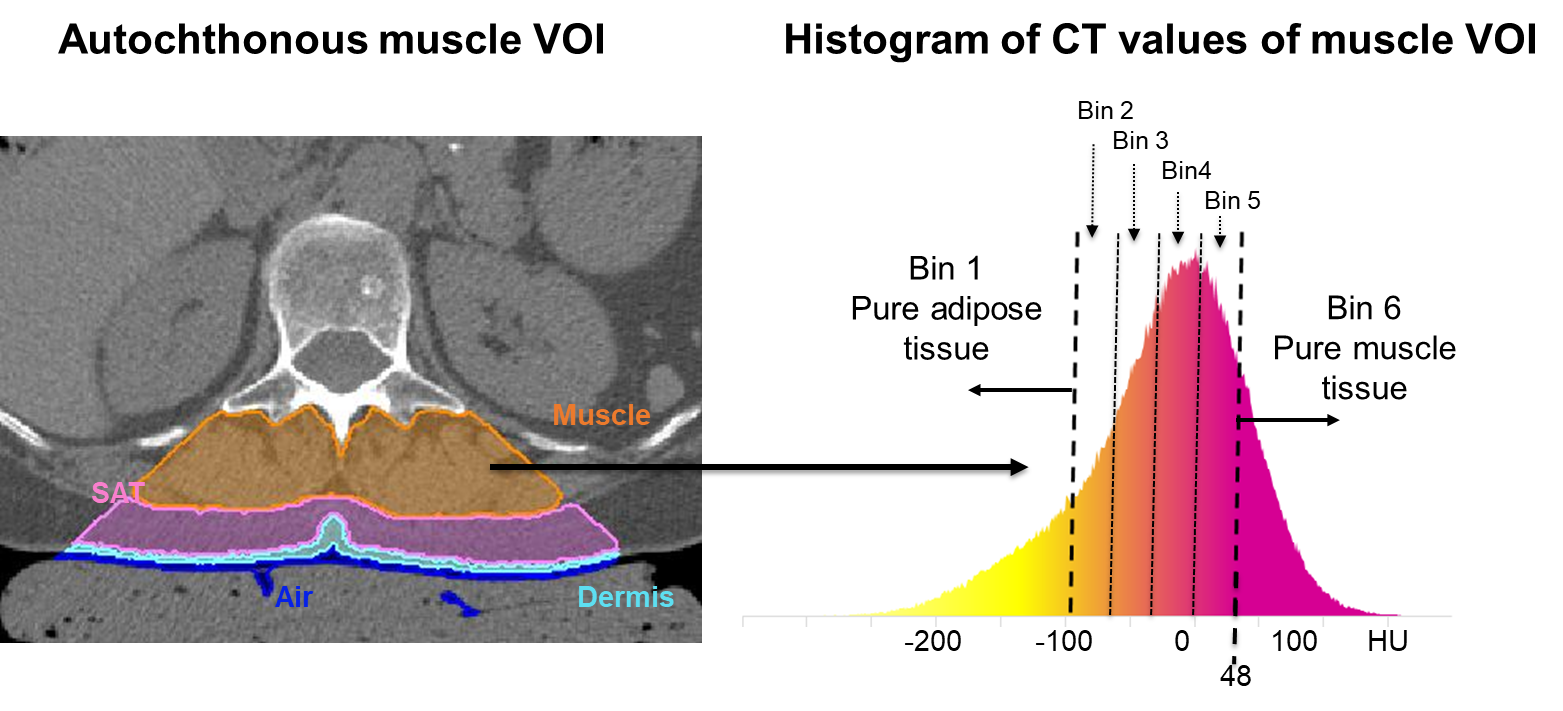


# Tables

Table S 1: Overview of BMD/BMC/Vol and cortical thickness assessments

|  | Total vertebral body (tVB) | | | | Central cylinder (cCy) | | Mid cylinder (mCy) | Mid vert body (mVB) | Upper endplate (uE) | | | Lower endplate (lE) |
| --- | --- | --- | --- | --- | --- | --- | --- | --- | --- | --- | --- | --- |
|  | Integral | Trabecular | Cortical | Trabecular | | Cortical | Trabecular | Cortical | | Cortical | Cortical | |
| BMD | X | X | X | X | | X |  | X | |  |  | |
| BMC | X | X | X | X | | X | X | X | |  |  | |
| Volume | X | X | X | X | | X |  | X | |  |  | |
| Thickness |  |  | X |  | |  |  | X | | X | X | |

Table S 2: Women: Predictors that remained in the individual models after binary logistic regression. Note: age and BMI were always retained in the models

|  | Beta | SE | p |
| --- | --- | --- | --- |
| **S1- BMD** |  | | |
| Age | 0.110 | 0.146 | 0.454 |
| BMI | 0.071 | 0.143 | 0.621 |
| BMD_Int_tVB | -0.543 | 0.183 | 0.003 |
| Thick_Cort_LE | -0.404 | 0.201 | 0.044 |
| **S2 Texture** |  | | |
| Age | 0.264 | 0.138 | 0.055 |
| BMI | 0.301 | 0.168 | 0.074 |
| Trab_gInhomo | -0.729 | 0.206 | 0.000 |
| **S3 Muscle** | * | | |

*no predictors of S3 remained in the model in addition to age and BMI

Table S 3: Men: Predictors that remained in the individual models after binary logistic regression. Note: age and BMI were always retained in the models

|  | Beta | SE | | p |
| --- | --- | --- | --- | --- |
| **S1 BMD** |  | | | |
| Age (SD) | 0.25 | 0.21 | | 0.23 |
| BMI (SD) | -0.05 | 0.23 | | 0.84 |
| BMD_Int_tVB | -0.72 | 0.26 | | 0.01 |
| **S2 Texture** |  | | | |
| Age (SD) | 0.458 | 0.213 | 0.032 | |
| BMI (SD) | -0.877 | 0.359 | | 0.015 |
| Trab_lAniso | 0.908 | 0.362 | | 0.012 |
| Trab_Entrop | 0.450 | 0.249 | | 0.071 |
| **S3 Muscle** |  | | | |
| Age (SD) | 0.381 | 0.212 | | 0.072 |
| BMI (SD) | -0.216 | 0.240 | | 0.368 |
| M_gAniso_Bin6 | 0.496 | 0.230 | | 0.031 |

Table S 4: Frequency in percent of predictors that remained significant in the logistic regression models after resampling 1000 datasets in the bootstrap analysis. Age and BMI always remained in the model regardless of their significance.

| **Women** | | **Men** | |
| --- | --- | --- | --- |
| **Predictor** | **Frequency** | **Predictor** | **Frequency** |
| Age | 100 | Age | 100 |
| BMI | 100 | BMI | 100 |
| Thick_Cort_lE | 77.3 | Weight | 40.2 |
| BMD_Trab_mCy | 26.1 | Thick_Cort_lE | 35.0 |
| BMC_Int_tVB | 25.5 | Thick_Cort_mVB | 28.3 |
| BMD_Int_tVB | 24.6 | Thick_Cort_uE | 28.1 |
| BMD_Cort_mVB | 24.2 | BMD_Trab_mCy | 25.5 |
| BMD_Trab_tVB | 16.4 | BMD_Cort_tVB | 25.2 |
| Thick_Cort_mVB | 14.9 | Height | 22.7 |
| BMC_Trab_tVB | 14.5 | BMD_Int_tVB | 21.3 |
| Thick_Cort_uE | 14.5 | BMD_Trab_tVB | 19.5 |
| Height | 13.3 | BMC_Trab_tVB | 19.4 |
| BMD_Trab_cCy | 12.2 | BMC_Int_tVB | 18.0 |
| Vol_Cort_mVB | 11.2 | BMD_Cort_mVB | 16.4 |
| Weight | 11.1 | BMD_Trab_cCy | 14.5 |
| BMD_Cort_tVB | 10.4 | Thick_Cort_tVB | 12.7 |
| BMC_Cort_tVB | 8.9 | Vol_Trab_tVB | 11.6 |
| BMC_Cort_mVB | 7.5 | BMC_Cort_tVB | 8.4 |
| Thick_Cort_tVB | 7.3 | Vol_Cort_tVB | 7.7 |
| Vol_Trab_tVB | 6.7 | Vol_Cort_mVB | 7.5 |
| Vol_Cort_tVB | 6.1 | Vol_Int_tVB | 7.3 |
| Vol_Int_tVB | 2.6 | BMC_Cort_mVB | 4.4 |

Table S 5: Performance of combinations of nested models tested by LRT in women with SQ2 and SQ3: Model 1, which is the base model, and Model 2, which represents the combined model.

| **Comparison of Nested Models** | | **Women** | | |
| --- | --- | --- | --- | --- |
| **Model 1** | **Model 2** | **DoF** | **LR χ2** | **p** |
| Age & BMI | S1 BMD | 351 | -12.4 | **<0.01** |
| S1 BMD | S2 Texture | 351 | 1.8 | n.s. |
| S1 BMD | S3 Muscle | 351 | 4.1 | **0.04** |

# References

1. McCloskey, E.V., A. Oden, N.C. Harvey, W.D. Leslie, D. Hans, H. Johansson, R. Barkmann, S. Boutroy, et al., *A Meta-Analysis of Trabecular Bone Score in Fracture Risk Prediction and Its Relationship to FRAX.* J Bone Miner Res, 2016. **31**(5): p. 940-8.

2. Lowitz, T., O. Museyko, V. Bousson, W.A. Kalender, J.D. Laredo, and K. Engelke, *Characterization of knee osteoarthritis-related changes in trabecular bone using texture parameters at various levels of spatial resolution-a simulation study.* Bonekey Rep, 2014. **3**: p. 615.

3. Lowitz, T., O. Museyko, V. Bousson, W.A. Kalender, J.D. Laredo, and K. Engelke, *A Digital Model to Simulate Effects of Bone Architecture Variations on Texture at Spatial Resolutions of CT, HR-pQCT, and muCT Scanners.* J Med Eng, 2014. **2014**: p. 946574.
